# Prognostic value of the pretreatment systemic immune-inflammation index in patients with prostate cancer: a systematic review and meta-analysis

**DOI:** 10.1186/s12967-023-03924-y

**Published:** 2023-02-04

**Authors:** Linghao Meng, Yujia Yang, Xu Hu, Ruohan Zhang, Xiang Li

**Affiliations:** 1 Institute of Urology, Department of Urology, West China Hospital, Sichuan University, Chengdu, 610041 China; 2 West China School of Medicine, Sichuan University, Chengdu, 610041 China; 3 West China School of Public Health and West China Fourth Hospital, Sichuan University, Chengdu, 610041 China

**Keywords:** Prostate cancer, Systemic immune-inflammation index, Survival, Prognosis, Meta-analysis

## Abstract

**Background:**

The systemic immune-inflammation index (SII) is a novel biomarker to predict the prognosis of some malignant tumors based on neutrophil, platelet, and lymphocyte counts. Evidence is scarce about the prognostic value of SII for prostate cancer patients. This systematic review and meta-analysis was conducted to explore the prognostic value of the SII in prostate cancer.

**Methods:**

The PubMed, Embase, Web of Science, and Cochrane Library (CENTRAL) databases were searched to determine eligible studies from inception to August 15, 2022. Hazard ratios (HRs) with 95% confidence intervals (CIs) were extracted to pool the results. Statistical analyses were conducted by using Stata 17.0 software.

**Results:**

A total of 12 studies with 8083 patients were included. The quantitative synthesis showed that a high SII was related to poor overall survival (OS) (HR = 1.44, 95% CI 1.23–1.69, *p* < 0.001). Furthermore, a subgroup analysis showed that a high SII was associated with poor OS in the groups of any ethnicity, tumor type, and cutoff value. An increased SII was also associated with inferior progression-free survival (PFS) (HR = 1.80, 95% CI 1.27–2.56, *p* = 0.001). In the subgroup analysis, a high SII value was related to poor PFS in Asian patients (HR = 4.03, 95% CI 1.07–15.17, *p* = 0.04) and a cutoff value > 580 (HR = 1.19, 95% CI 1.04–1.36, *p* = 0.01).

**Conclusion:**

Based on the current evidence, a high pretreatment SII may be associated with poor OS and PFS. The SII may serve as an important prognostic indicator in patients with prostate cancer. More rigorously designed studies are needed to explore the SII and the prognosis of prostate cancer.

## Introduction

Prostate cancer (PCa) is one of the most common cancers in the urinary system, with an estimated 1,414,259 new cases and 375,304 deaths worldwide in 2020 [1]. Advanced age, genetic alteration, diet, metabolism, and sexual behaviors are considered risk factors for PCa [2, 3]. Prostate-specific antigen (PSA) level, TNM stage, and pathological Gleason score were used as the main evidence for therapies in the clinical practice of PCa patients [4, 5]. For localized or locally advanced PCa, radical prostatectomy (RP) and radiotherapy are effective therapies that are related to a positive prognosis [6–8]. Androgen deprivation therapy (ADT) combined with chemotherapy or new hormonal treatment can prolong the overall survival (OS) of metastatic prostate cancer patients [9–12]. However, most patients will progress to castration-resistant prostate cancer (CRPC) within 2–3 years after ADT, which is a more aggressive and critical stage [13]. Some clinicopathological characteristic systems, including the International Society of Urological Pathology prostate cancer grading and Gleason grading system, might be powerful prognostic indicators for PCa [14, 15]. Moreover, genomics and proteomics biomarkers also have potential prognostic value in PCa patients [16, 17]. However, the systems above still have deficiencies that may influence treatment and patient care [18]. Therefore, considering that the prognosis of PCa patients is still unsatisfactory, reliable biomarkers should be developed to assist in clinical decisions regarding diagnosis, treatment, and prognosis.

Tumor-associated immune reactions in the tumor microenvironment (TME) act as immunological surveillance and antitumor immune responses, which are closely related to the prognosis of patients [19]. Thus, some immune inflammatory indicators might become potential parameters for tumor diagnosis and prognosis. Recently, some hematology indicators, including the neutrophil-lymphocyte ratio (NLR), platelet-lymphocyte ratio (PLR), and lymphocyte-monocyte ratio (LMR), have been reported to have predictive value for PCa [20, 21]. These indicators have convenient and rapid characteristics in clinical practice as favorable prognosis predictors [20]. The systemic immune-inflammation index (SII) is an effective parameter to show the systemic immune and inflammation condition [22]. SII is defined as neutrophils × platelets/lymphocytes in peripheral blood and has been proven to be associated with poor prognosis in many malignant solid tumors [22–25].

Although SII integrated three types of hematology indicators, the results for the prognostic value of SII in PCa patients were not robust. Rajwa et al. [26] explored the effect of SII in patients treated with RP and found that SII was associated with adverse clinicopathological characteristics and OS. However, another study conducted in 2020 showed no statistical significance of survival [27]. The reasons may be related to the differences in sample size, the characteristics of patients, and therapies. Therefore, we conducted this systematic review and meta-analysis to investigate the prognostic value of SII in PCa patients, aiming to predict the prognostic factors of PCa more precisely.

## Methods

We performed this systematic review and meta-analysis following the Preferred Reporting Items for Systematic Reviews and Meta-analyses (PRISMA) guidelines [28]. This study has been registered in PROSPERO (CRD42022353480).

### Search strategy

We searched the PubMed, Embase, Web of Science, and Cochrane Library (CENTRAL) databases to identify eligible studies up to August 15, 2022. We applied the following terms: (prostatic neoplasm* OR prostate neoplasm* OR prostate cancer* OR prostatic cancer* OR PCa) AND (systemic immune-inflammation index OR SII). MeSH and free text terms were used to identify the eligible literature. We manually screened the references of the included studies to identify potentially eligible studies.

### Inclusion and exclusion criteria

The included studies met the following criteria: (1) randomized controlled trials or observational studies; (2) patients were pathologically diagnosed with PCa; (3) studies exploring the relationship between pretreatment SII and prognosis in PCa patients; (4) studies reporting the cutoff value of SII; (5) studies providing hazard ratios (HRs) with 95% confidence intervals (CIs); and (6) studies reporting survival outcomes, including OS, progression-free survival (PFS), or cancer-specific survival (CSS).

The following studies were excluded: (1) non-English language; (2) duplicate articles; (3) reviews, letters, case reports, protocols, conference abstracts, and any article without full text; (4) in vitro or animal experiments; and (5) studies that did not provide sufficient data.

### Data extraction and quality assessment

Two authors (LM and YY) independently extracted the data from the included studies. Disagreements were resolved by discussion with a third reviewer (XL). The following data were extracted: first author, publication year, country or region, study design, sample size, age of patients, tumor type, treatment methods, cutoff value of SII, analysis methods, follow-up period, survival outcomes, HRs, and 95% CIs. The Newcastle‒Ottawa quality assessment scale (NOS) was used to assess the risk of bias [29]. The NOS scale in observational studies included eight parts and ranged from 0 to 9. A score of no less than 7 was considered high quality [30].

### Statistical analysis

We synthesized the HRs and CIs to determine the prognostic value of SII in PCa patients. Pooled HR > 1 without 95% CI overlapping 1 suggested that a high SII was associated with poor prognosis of PCa patients. Cochran’s Q and Higgin’s I^2^ tests were used to assess the heterogeneity of the studies. When I^2^ > 50% or *p* < 0.10, we considered significant heterogeneity, and therefore, a random-effects model was used. We conducted a subgroup analysis to explore the potential sources of heterogeneity. Ethnicity, tumor type, treatment method, sample size, and cut-off value were considered to execute the subgroup analysis in order to explore the influence of confounding factors. Begg’s test and Egger’s test were performed to assess publication bias. If there were potential publication bias from the included studies, Duval and Tweedie nonparametric trim and fill procedure would be used to explore the effect of missing studies. Concrete details and interpretation were available in the reference [31]. We also conducted a sensitivity analysis by using eliminate one by one method to evaluate the robustness of the results. Stata 17.0 software (Stata Corp LP, College Station, TX, USA) was used for statistical analysis. A *P* < 0.05 (two-tailed) was considered statistically significant.

## Results

### Characteristics of the included studies

**Fig. 1:**
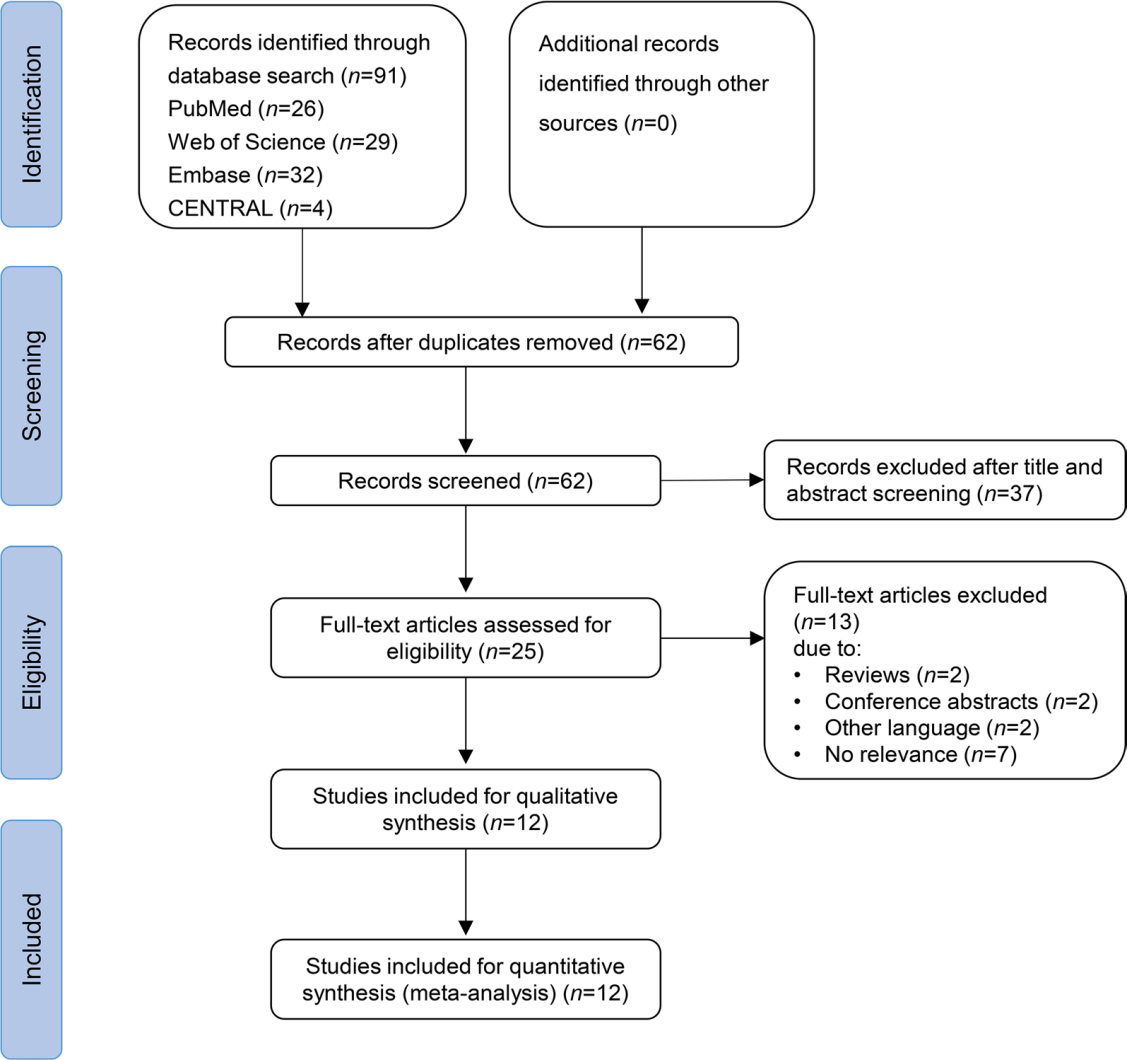
Flowchart of literature screening

The detailed flowchart of the study search and selection process is shown in Fig. 1. From the database, we identified 91 publications, and 62 articles were included after removing duplicates. After title and abstract screening, 25 publications were eligible for full-text screening. Then, we excluded 13 studies because 2 studies were reviews, 2 studies were conference abstracts, 2 studies were in other languages, and 7 studies had no relevance to the topic. Finally, 12 studies with 8083 patients were eligible for quantitative synthesis[26, 27, 32–41].

All of the included studies were retrospective and published between 2016 and 2022. Three studies were conducted in China [34, 37, 41], three in Italy [32, 33, 36], two in Germany [38, 39], one in Japan [35], one in Austria [27], and two in multiple centers [26, 40]. Regarding the tumor type, 8 studies focused on CRPC [27, 32–39], and the other 4 studies focused on other types of PCa [26, 39–41]. The cutoff values of SII ranged from 160 to 1091 (median 580). Ten studies reported the relationship between SII and OS [26, 27, 32–39], six reported PFS [26, 27, 34, 35, 40, 41], and only one reported CSS[26]. All of the included studies were considered high quality (no less than 7). More detailed characteristics are available in Table 1.

**Table 1:** Baseline characteristics of the included studies and methodological assessment

Author	Year	Country	Study design	Sample size	Age	Tumor type	Treatment methods	Cutoff value	Follow-up (months)	Survival outcome	Quality score
Bauckneht (a) [32]	2020	Italy	Retrospective	48	Median 75 (51–88)	mCRPC	Radium-223	1091	Median 10	OS	7
Bauckneht (b) [33]	2022	Italy	Retrospective	494	Median 74 (50–90)	mCRPC	Radium-223	769	Median 10.7	OS	8
Fan [34]	2018	China	Retrospective	104	Median 72.0 (65.3–77.0)	mCRPC	Docetaxel + abiraterone	200	Median 20.2	OS, PFS	8
Kobayashi [35]	2022	Japan	Retrospective	144	Median 71 (66–76)	CRPC	Docetaxel (+abiraterone/enzalutamide)	636	NR	OS, PFS	8
Lolli [36]	2016	Italy	Retrospective	230	Median 74 (45–90)	mCRPC	Docetaxel + abiraterone	535	Median 29	OS	8
Man [37]	2019	China	Retrospective	179	Median 70 (51–88)	mCRPC	Docetaxel	535	Median 24	OS	8
Neuberger (a) [39]	2022	Germany	Retrospective	36	Median 64 (60–70)	mHSPC	Docetaxel	801	NR	OS	8
Neuberger (b) [38]	2022	Germany	Retrospective	118	Median 72 (65–76)	mCRPC	Docetaxel	160	NR	OS	8
Rajwa (a) [40]	2021	Multicenter	Retrospective	6039	Median 61 (57–66)	nmPCa	RP	620	Median 44	PFS	8
Rajwa (b) [26]	2021	Multicenter	Retrospective	214	Median 69 (64, 72)	nmPCa	RP	730	Median 25.3	OS, PFS, CSS	8
Stangl-Kremser [27]	2020	Austria	Retrospective	186	Median 68.8 (64.6–75.0)	CRPC	Docetaxel	200	NR	OS, PFS	8
Wang [41]	2022	China	Retrospective	291	Median 66.13 ± 6.05	Localized PCa	RP	528.54	Median 48	PFS	8

*mCRPC* metastatic castration-resistant prostate cancer, *CRPC* castration-resistant prostate cancer, *mHSPC* metastatic hormone-sensitive prostate cancer, *nmPCa* nonmetastatic prostate cancer, *PCa* prostate cancer, *RP* radical prostatectomy, *NR* not reported, *OS* overall survival, *PFS* progression-free survival, *CSS* cancer-specific survival

### Prognostic significance of the SII for OS in prostate cancer

**Fig. 2:**
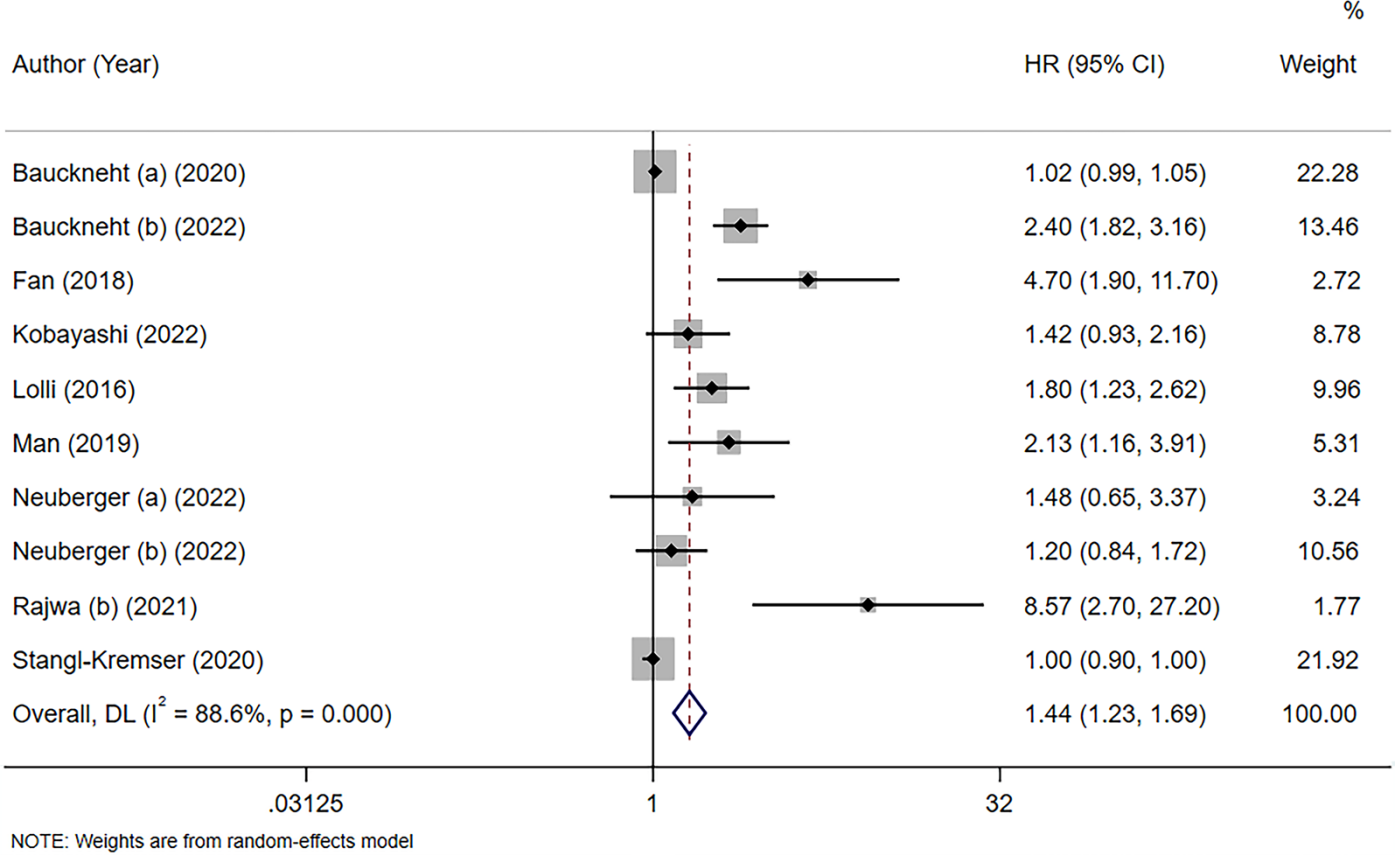
Forest plot showing the association between the systemic immune-inflammation index (SII) and overall survival (OS) in prostate cancer (PCa)

Ten studies including 1753 patients focused on the association between SII and OS in patients with PCa [26, 27, 32–39]. The pooled HR showed that elevated preoperative SII was significantly associated with poor OS (HR = 1.44, 95% CI 1.23–1.69, *p* < 0.001). Significant heterogeneity was detected in the included studies (I^2^ = 88%, *p* < 0.001) **(**Fig. 2**)**. We performed subgroup analysis based on ethnicity, tumor type, treatment method, sample size, and cutoff value of SII. A high SII was significantly associated with worse OS in the subgroups of any ethnicity, tumor type, and cutoff value. A high SII was associated with worse OS in patients treated with chemotherapy + androgen receptor targeting agents (ARTA) (HR = 1.94, 95% CI 1.03–3.67, *p* = 0.04) and in studies with a sample size > 200 (HR = 2.54, 95% CI 1.55–4.16, *p* < 0.001). The details are available in Table 2.

**Table 2:** Subgroup analyses of overall survival (OS) and progression-free survival (PFS)

Outcome	Subgroups	No. of studies	Effects model	HR (95% CI)	*p*	Heterogeneity	
						I^2^ (%)	*p*
OS	All	10	Random	1.44 (1.23, 1.69)	0.001	88.0	0.001
Ethnicity	Asian	3	Random	2.18 (1.19, 4.00)	0.012	65.3	0.056
	Nonasian	6	Random	1.26 (1.08, 1.46)	0.002	89.5	0.000
Tumor type	CRPC	8	Random	1.37 (1.17, 1.60)	0.000	89.3	0.000
	mPCa	7	Random	1.75 (1.17, 2.60)	0.006	90.4	0.000
Treatment	Radiotherapy	2	Random	1.55 (0.67, 3.58)	0.308	97.3	0.000
	Chemotherapy	5	Random	1.39 (1.00, 1.93)	0.052	72.0	0.006
	Chemotherapy + ARTA	3	Random	1.94 (1.03, 3.67)	0.041	69.3	0.038
Sample size	≤ 200	7	Random	1.09 (0.98, 1.22)	0.098	71.5	0.002
	> 200	3	Random	2.54 (1.55, 4.16)	0.000	70.1	0.035
Cut off value	≤ 580	5	Random	1.61 (1.07, 2.43)	0.022	84.9	0.000
	> 580	5	Random	1.85 (1.07, 3.19)	0.028	82.4	0.000
PFS	All	6	Random	1.80 (1.27, 2.56)	0.001	92.0	0.000
Ethnicity	Asian	3	Random	4.03 (1.07, 15.17)	0.039	93.2	0.000
Tumor type	CRPC	3	Random	2.30 (0.88, 6.00)	0.089	95.5	0.000
	nmPCa	3	Random	1.78 (0.94, 3.35)	0.075	86.3	0.001
Treatment	Chemotherapy	2	Random	1.31 (0.71, 2.41)	0.389	84.9	0.010
	Chemotherapy + ARTA	2	Random	3.04 (0.22, 42.53)	0.408	96.7	0.000
	RP	3	Random	1.78 (0.94, 3.35)	0.075	86.3	0.001
Sample size	≤ 200	3	Random	2.30 (0.88, 6.00)	0.089	95.5	0.000
	> 200	3	Random	1.78 (0.94, 3.35)	0.075	86.3	0.001
Cut off value	≤ 580	3	Random	3.65 (0.72, 18.37)	0.116	96.7	0.000
	> 580	3	Random	1.19 (1.04, 1.36)	0.010	0.0	0.599

*HR* hazard ratio, *CI* confidence interval, *OS* overall survival, *PFS* progression-free survival, *CRPC* castration-resistant prostate cancer, *mPCa* metastatic prostate cancer, *nmPCa* nonmetastatic prostate cancer, *ARTA* androgen receptor targeting agents, *RP* radical prostatectomy

### Prognostic significance of the SII for PFS in prostate cancer

**Fig. 3:**
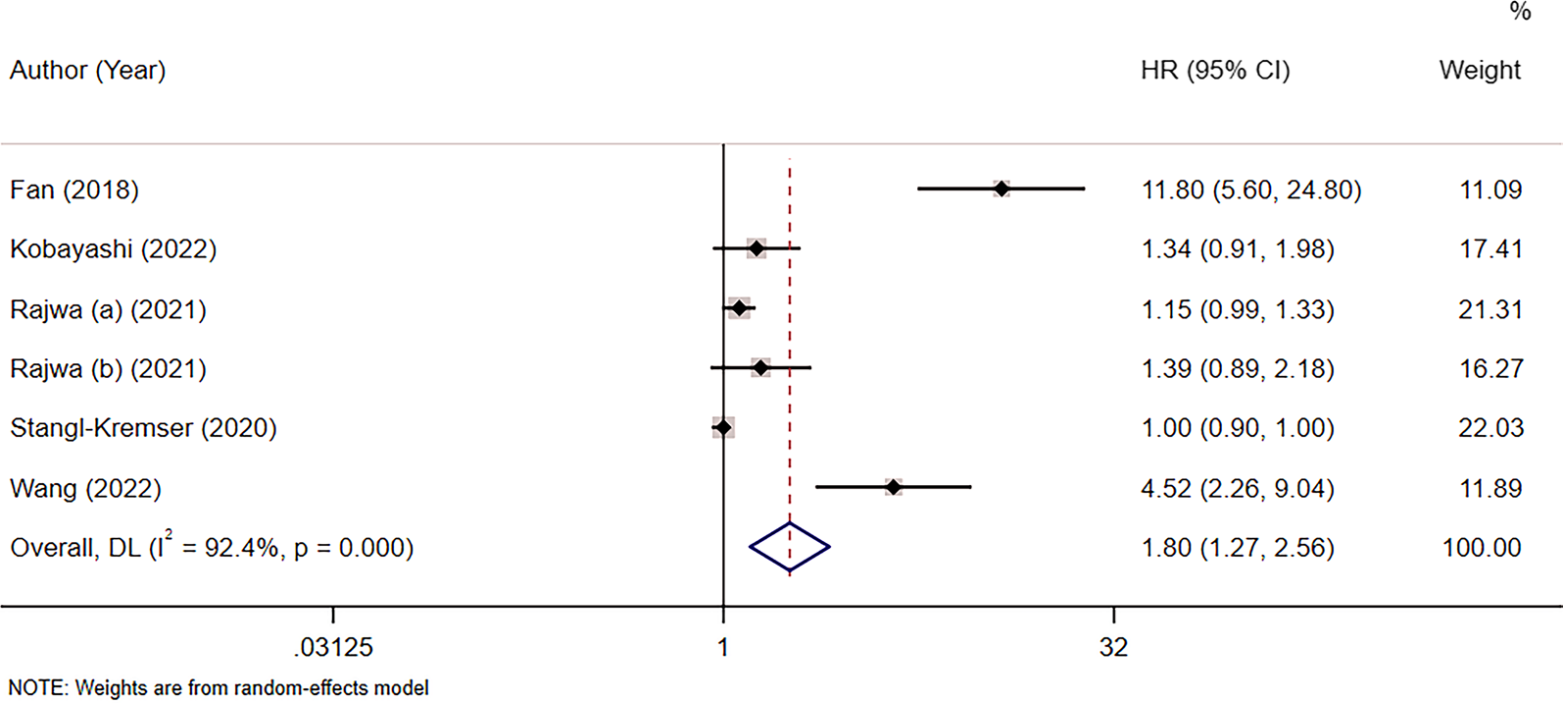
Forest plot showing the association between the systemic immune-inflammation index (SII) and progression-free survival (PFS) in prostate cancer (PCa)

Six studies comprising 6978 patients focused on the relationship between SII and PFS in patients with PCa [26, 27, 34, 35, 40, 41]. The pooled analysis showed that the increased preoperative SII was a prognostic predictor of PFS, with HR = 1.80, 95% CI 1.27–2.56, *p* = 0.001. A random-effects model was used because of the significant heterogeneity (I^2^ = 92%, *p* < 0.001). In the subgroup analysis, a high SII value was significantly associated with poor PFS in Asian patients and a cutoff value > 580, with HR = 4.03, 95% CI 1.07–15.17, *p* = 0.04 and HR = 1.19, 95% CI 1.04–1.36, *p* = 0.01, respectively **(**Fig. 3; Table 2**)**.

### Publication bias

**Fig. 4:**
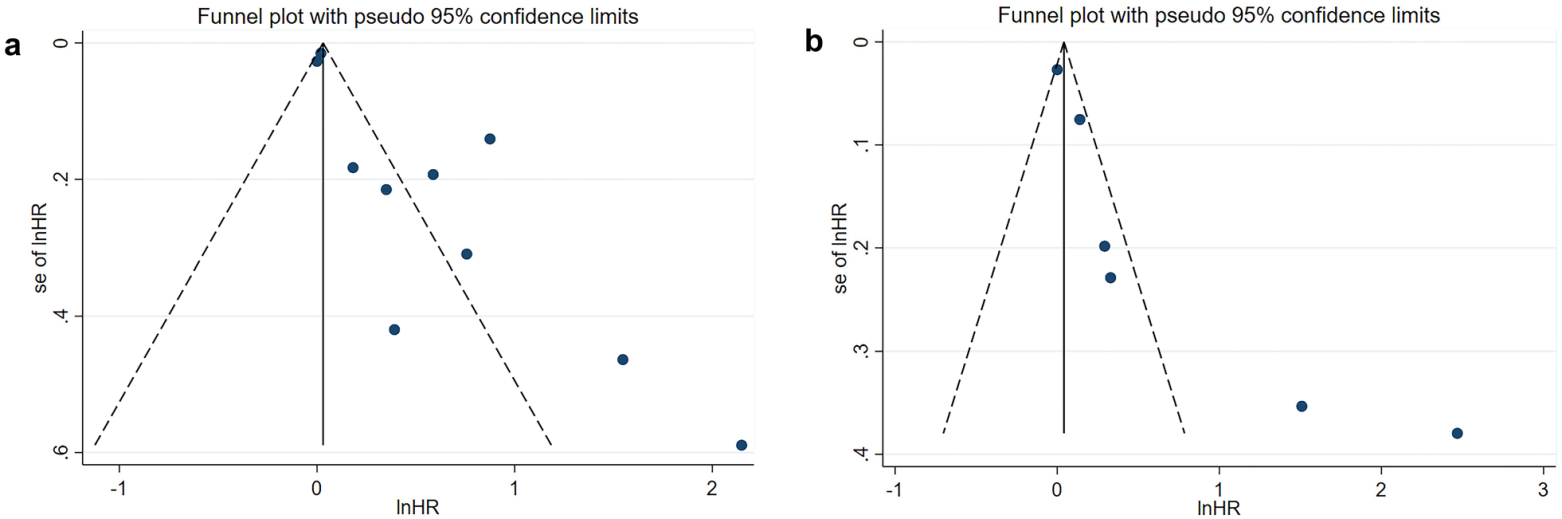
Publication bias assessment using funnel plots for overall survival (OS) and progression-free survival (PFS). **a** OS; **b** PFS

**Fig. 5:**
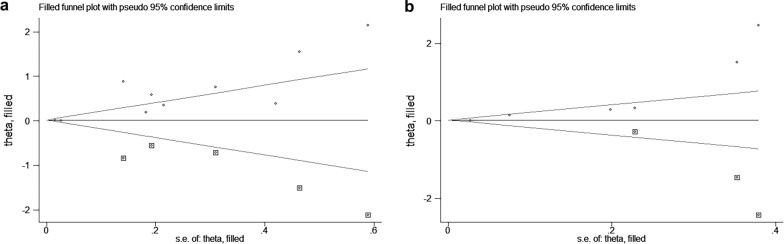
Duval and Tweedie nonparametric trim and fill method for overall survival (OS) and progression-free survival (PFS). **a** OS; **b** PFS

We used Begg’s test and Egger’s test to analyze the publication bias of OS and PFS of the included studies. The results of Begg’s test showed no significant publication bias in the meta-analysis (OS: *p* = 0.074; PFS: *p* = 0.133). However, Egger’s test showed significant publication bias (OS: *p* = 0.003; PFS: *p* = 0.026), and the asymmetric funnel plot showed the potential publication bias of the meta-analysis **(**Fig. 4**)**. Therefore, we conducted the Duval and Tweedie nonparametric trim and fill procedure to evaluate the effect of the potential missing studies [42]. After filling in the possible missing studies, the results indicated that the meta-analysis may not be stable **(**Fig. 5**)**.

### Sensitivity analysis

**Fig. 6:**
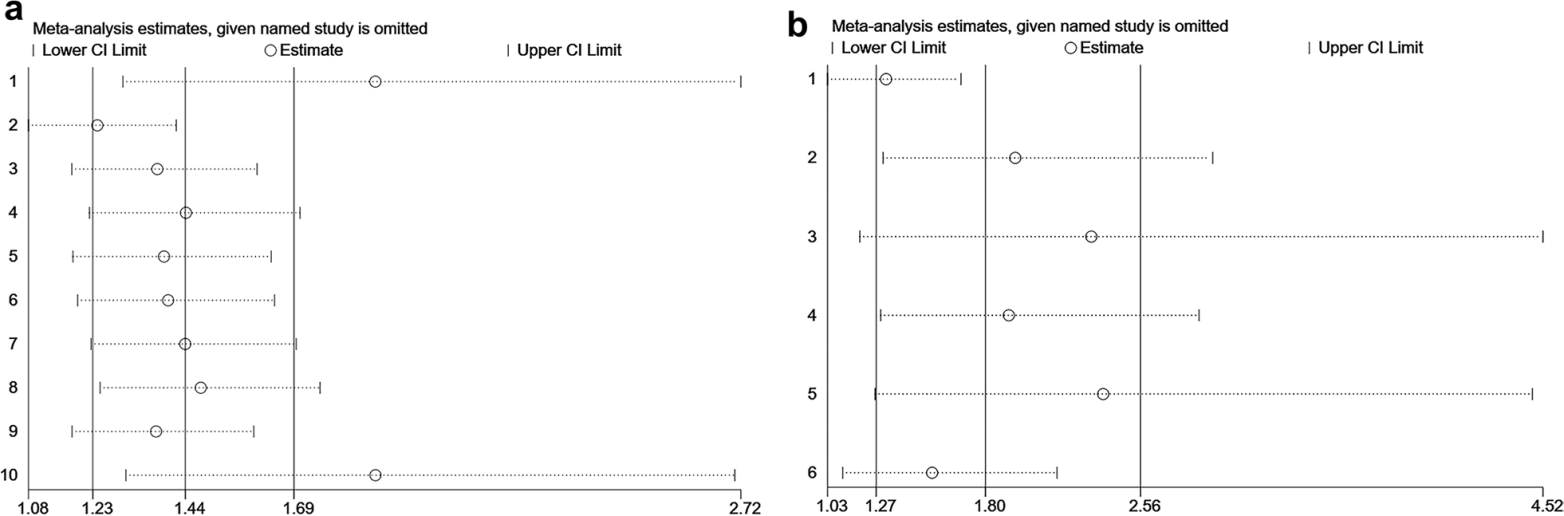
Sensitivity analysis using the leave-one-out test for overall survival (OS) and progression-free survival (PFS). **a** OS; **b** PFS

Sensitivity analysis was used to assess the robustness of the meta-analysis. The leave-one-out test showed that no single study influenced the results, indicating that the results of the meta-analysis were stable and reliable **(**Fig. 6**)**.

## Discussion

Presently, the surveillance of progression and estimation of prognosis in patients with PCa mainly rely on conventional clinicopathological variables, such as Gleason scores and PSA, which could partly reflect cancer behavior in biology but may not represent the actual status of PCa. In recent years, SII, an index based on blood tests, has shown great potential as a complementary item for the prediction of survival in patients with malignancies. The impact of SII on the prognosis of solid tumors, including breast cancer [43], pancreatic cancer [44], lung cancer [45], and cervical cancer [46], as well as solid tumors of the urinary system, such as bladder cancer [47], has been reported in many studies. Several studies of SII have been summarized in such systematic reviews and meta-analyses, which provided penetrating insight into the association between SII and solid tumors, such as colorectal cancer [48], uterine cervical cancer [49], breast cancer [24], and pancreatic cancer [50]. The majority of these studies showed that a high SII is associated with poor prognosis in patients with such cancers. Nonetheless, the prognostic value of SII in PCa patients is still unclear. Qi et al. [51]conducted a meta-analysis including 10 studies with PCa, and they focused on the association of SII and nmPCa/mCRPC. They found there was no significant association between SII and PFS in mCRPC patients. Besides, the subgroup analysis in the study only concentrated on ethnicity, cutoff value and sample size, not including treatment methods. In our study, syncretic data of PFS and BFS was analyzed simultaneously because of similar definitions of these two concepts mentioned in the studies included. Furthermore, the treatment measure is one of the most meaningful emphases in prediction of survival outcomes in patients with PCa. To discover the accurate effect of SII on the prognosis of PCa, we performed a meta-analysis, including 12 articles and 8083 patients, to investigate the association between SII status and the prognosis of PCa.

SII is based on the quantity of platelets (P; × 10^9^/l), neutrophils (N; × 10^9^/l) and lymphocytes (L; × 10^9^/l) in peripheral blood using the following formula: SII = P × N/L [52]. All the data used for calculation could be acquired from routine blood tests, which means that investigators could collect and analyze SII data without any difficulty. Platelets, neutrophils, and lymphocytes are all significant components of the TME that affect tumor cell proliferation and invasion in multiple aspects [53]. Such studies have already researched the association between immune cells and PCa. A meta-analysis by Guan et al. noted that the NLR and PLR were effective biomarkers for predicting prognosis in metastatic CRPC patients [54]. Platelets mainly regulate blood coagulation and hemostasis. The asymmetric status of platelets contributes to cancer promotion and progression, directly causing thrombocytosis in patients with cancer [55]. Present cell research has already indicated that platelets enhance the invasion of androgen receptor-negative PCa cells via increased matrix metalloproteinase expression [56]. In such a clinical trial, the application of antiplatelet or anticoagulant therapy and platelet counts were associated with freedom from biochemical failure and distant metastasis in PCa patients who received primary radiotherapy [57]. Neutrophils have been proven to play an important role in antitumor and protumor processes by regulating the immune response against tumor cells [58], which attenuates antitumor immunity, reinforces tumor cell survival, and increases angiogenesis [59]. In addition to the proven immune function of neutrophils, molecules in the granules of neutrophils, including neutrophil gelatinase-associated lipocalin, were proven to be stabilizers of matrix metalloproteinase 9 [60], which is involved in the degradation of the extracellular matrix and plays a significant role in metastasis and cancer progression. Lymphocytes, especially T-lymphocytes, are known to be an effective tool for the antitumor immune response [61]. Promoting infiltration of T-lymphocytes in the TME by downregulating immunosuppressive cytokines (IL10, IL6) has been reported as an effective way to enhance antitumor immunity [62]. According to several publications, infiltration of more lymphocytes in the TME is correlated with better survival of cancer patients [63], which indicates that lymphocyte count is associated with the immune escape of cancer cells. Indicators associated with such immune cells, including NLR, PLR, and LMR, have already been used as predictive indicators of PCa [64]. These ratios could also help doctors predict upgrading of Gleason score in the assessment of low-risk PCa when considering patients for active surveillance [65]. Some studies explored the predictive effect of PLR, NLR, and SII in prostate cancer simultaneously, indicating that SII could reflect the association between systemic inflammation and cancer more objectively than other indexes [37]. In recent years, many studies have been carried out to determine the safety and effectiveness of novel immunotherapies, such as cancer vaccines as a precaution for mucosal cancers by enhancing tissue resident memory T cells [66] and immune checkpoint inhibitors, which have a corrosive effect on tumor cells through stimulation of antitumor immunity [67].

A high SII, which signifies high neutrophil or platelet counts and/or low lymphocyte counts, is correlated with poor prognosis in PCa patients and may follow the mechanism mentioned above. Apparently, our results showed that poor survival outcomes of PCa patients were associated with high SII regardless of tumor type, ethnicity and cutoff value, consistent with several previous studies focused on SII and other solid tumors [50, 68, 69]. Our results indicated that the SII also played a crucial role in the prediction of prognosis in PCa, which proved the tight connection between the progression of PCa and the status of the immune system. Novel antitumor medicines of therapeutic targets associated with the immune system, including PD-1 and PD-L1, have already been applied in clinical practice. Recent studies have concentrated on several novel small molecule inhibitors based on interference with the pathway of PD-1 and PD-L1 by blocking the direct interaction between PD-1 and PD-L1, inhibiting the transcription and translation of PD-L1, and promoting the degradation of the PD-L1 protein [70]. For PCa, tremelimumab plus durvalumab was safe and well tolerated in patients with chemotherapy-naïve metastatic CRPC to bone [71], but the effectiveness of immune checkpoint therapy (ICT) still needs more large-scale studies. Components of peripheral blood may have growing prognostic value and great potential as therapeutic targets for PCa in the future. Except for classical biomarkers used for the prognosis and diagnosis of PCa, such as PSA, a growing number of indicators that could be conveniently obtained from simple blood tests are used for the prediction of tumorigenesis and tumor progression. Two of studies (Fan. et al. and Kobayashi. et al.) included in our subgroup analysis of SII and PFS in PCa patients found no significant association between PFS and classical parameters (PSA and biopsy Gleason score) of PCa patients but SII showed specific effect on PFS. This indicated the supplementary function and necessity of SII in predicting the PFS of PCa patients. The combination of this information would help doctors predict the prognosis of patients with PCa more precisely.

As we described previously, advantages of SII as a marker for predicting the prognosis of PCa including (1) SII partly represents the actual status of immune system in PCa patients. (2) SII includes more items of immune cells than NLR and PLR which had been already researched in survival outcomes of PCa. (3) All the data used for calculation of SII could be acquired from routine blood tests, which means that investigators could collect and analyze SII data without any difficulty. (4) SII may have the potential of being a decisive factor for immunotherapy. Limitations of this meta-analysis are as follows: (1) The cutoff values of SII are variable across the studies included in the analysis, which may cause substantial heterogeneity between the studies included. The heterogeneity might confine its application in clinical practice; hence, more credible evidence is needed to identify the optimal cutoff values of SII. (2) All the studies in this meta-analysis were retrospective, which may cause selection bias. Evaluation of the effect of the potential missing studies through the Duval and Tweedie nonparametric trim and fill procedure indicated that publication bias may exist in studies included in this meta-analysis. (3) This meta-analysis only included 12 studies and 8083 patients. Sample size of studies included in this meta-analysis is of great discrepancy. The comparatively small sample size caused relatively insufficient reliability of this study. Population-based studies are necessary in the future to provide profound and accurate evidence for this subject.

## Conclusion

This systematic review and meta-analysis suggested that a high SII value was significantly associated with poor OS and PFS in patients with PCa. The SII may serve as an independent effective prognostic indicator for PCa. Furthermore, more rigorously designed studies should be conducted for use in clinical practice.

## Data Availability

All data generated or analyzed during this study are included in this published article [and its supplementary information files].

## Abbreviations

PCa: Prostate cancer
PSA: Prostate-specific antigen
RP: Radical prostatectomy
ADT: Androgen deprivation therapy
OS: Overall survival
CRPC: Castration-resistant prostate cancer
TME: Tumor microenvironment
NLR: Neutrophil-lymphocyte ratio
PLR: Platelet-lymphocyte ratio
LMR: Lymphocyte-monocyte ratio
SII: Systemic immune-inflammation index
PRISMA: Preferred Reporting Items for Systematic Reviews and Meta-analyses
HRs: Hazard ratios
CIs: Confidence intervals
PFS: Progression-free survival
CSS: Cancer-specific survival
NOS: Newcastle–Ottawa quality assessment scale
ARTA: Androgen receptor targeting agents
ICT: Immune checkpoint therapy
